# Host preference and specialization in the genus *Aphanomyces* (Oomycetes) from molecular and interaction network insights

**DOI:** 10.1038/s41598-026-44513-5

**Published:** 2026-03-19

**Authors:** Gloria Casabella-Herrero, Laura Martín-Torrijos, Sergio Pérez-Ortega, Javier Diéguez-Uribeondo

**Affiliations:** Mycology Department, Real Jardín Botánico CSIC, Plaza Murillo 2, 28014 Madrid, Spain

**Keywords:** *Aphanomyces*, Invasive species, Pathogen, Evolution, Host preference, Specialization, Ecology, Ecology, Evolution, Plant sciences

## Abstract

*Aphanomyces* (Saprolegniales, Oomycetes) includes some of the most economically and environmentally devastating pathogens of plants and freshwater animals. Despite research focused on its pathogenicity and dispersal, key biological questions, such as host range and specialization, remain poorly understood. In this study, we investigated the host preference and specialization within *Aphanomyces*. First, we delineated species boundaries employing delimitation algorithms based on 261 representative sequences of the nuclear ribosomal internal transcriber spacer (nrITS) from 261 samples of 20 described species and several unidentified taxa. Our results suggested that *Aphanomyces* diversity was previously underestimated, since we identified 14 putative new species. Furthermore, we compiled an interaction database of 1221 host records. Network analyses revealed *Aphanomyces* as a highly specialized group mainly targeting two clusters of hosts: freshwater animals or plants in wet terrestrial soils. In addition, pathogenic *Aphanomyces* species appeared to be highly specialized. This study represents the first investigation encompassing the full diversity of *Aphanomyces* species, not only economically relevant species. Our results set the basis to better understand the evolutionary history of host specialization of harmful oomycete pathogens.

**Supplementary Information:**

The online version contains supplementary material available at 10.1038/s41598-026-44513-5.

## Introduction

The class Oomycetes are a diverse group of fungal-like organisms related to diatoms and brown algae^1,2^ that include some of the most economically and ecologically significant pathogens affecting plants and animals worldwide^3,4^. Among these, the genus *Aphanomyces* (Verrucalvaceae, Saprolegniales) contains highly specialized pathogens responsible for severe diseases in freshwater animals and plants. Notably, *Aphanomyces astaci* causes the crayfish plague, which threatens the extinction of Eurasian freshwater crayfish species^5^, while *Aphanomyces invadans* is the etiological agent of Epizootic Ulcerative Syndrome (EUS) in fish, a disease that devastated aquaculture in the Asia–Pacific region during the 1990s and has since spread to North America, Australia, and Africa^6^. In agriculture, *Aphanomyces euteiches* accounts for up to 80% of crop losses in legumes^7,8^, and *Aphanomyces cladogamus* and *Aphanomyces cochlioides* cause substantial damage to crops in the Fabaceae, Solanaceae, Chenopodiaceae, and Amaranthaceae families^7,9^.

Despite extensive research on the pathogenic species of *Aphanomyces*, key questions regarding the evolution, ecology and host specificity of these organisms remain unresolved. Thus, the genus is poorly understood in terms of host-range diversity, a gap attributed to (i) difficulties in species identification, (ii) the absence of a robust molecular taxonomy, and (iii) the limited number of phylogenetic studies. Phylogenetic analysis on oomycetes have questioned the monophyly of *Aphanomyces*, due to the occurrence of species from the genera *Plectospira*^15,16^ and *Phragmosporangium*^17^ (Verrucalvaceae) nested within this genus. Specifically, the only major phylogenetic analysis dedicated to the genus *Aphanomyces* to date identified three distinct ecological lineages, suggesting ecological and host specialization^10^. However, this work remains underexplored, and no comprehensive review of host breadth across the genus has been conducted.

Understanding host specialization is critical for advancing our knowledge of *Aphanomyces* biology, pathogenicity and evolutionary strategies^11,12^. Interaction network analyses, which have been successfully employed in other fungal pathogens to explore host–pathogen dynamics^13,14^, have yet to be applied to *Aphanomyces*. This represents a significant opportunity to deepen our understanding of host preference and specialization in this genus.

In this study, we aim to address these gaps by (i) constructing an updated phylogeny of *Aphanomyces* via a molecular species delimitation analyses that also includes, for the first time, key phylogenetically related genera such as *Phragmosporangium* and *Plectospira*^15–17^), and (ii) conducting an interaction network analysis based on an extensive review of habitat preference data from the literature and molecular databases. Our approach will provide new insights into the evolutionary and ecological forces driving host–pathogen interactions within *Aphanomyces* and offer a more comprehensive understanding of its ecological diversity and the evolutionary circumscription of these organism.

## Materials and methods

### DNA extraction, amplification and sequencing from cultures

A total of 51 single-spore isolates were selected from the oomycete culture collection of the Real Jardín Botánico-CSIC (RJB-CSIC), Madrid Spain. The selected single-spore isolates included 10 *Aphanomyces* species (*A. astaci, A. cochlioides, A. euteiches, A. frigidophilus, A. laevis, A. repetans, A. salsuginosus, A. sinensis, A. stellatus* and *Aphanomyces* sp.) and one isolate from the genus *Plectospira* (Table 1). We grew each of the selected cultures in peptone glucose liquid medium for 2 days at room temperature^18^. Each growing-culture was collected and transferred into a 2 ml tube and frozen at -80ºC prior to lyophilization using a BenchTop K (VirTis). The mycelium was mechanically disrupted using a TissueLysser II (QIAGEN)^19^ before DNA isolation following a CTAB-method^20^.

**Table 1:** GenBank accession numbers, strain name, geographic origin (if specified) and original species assignation of the 261 sequences from the nuclear ribosomal Internal Transcriber Spacer (nrITS) region of *Aphanomyces*, *Phragmosporangium* and *Plectospira* genera used in this study. Sequenced isolates from the Real Jardín Botánico (RJB-CSIC) culture collection of oomycetes are also included under the “CCRJB” strain name. Column “Putative species assignation” refers to the results of the species delimitation analyses (ABGD, ASAP, GMYC and bPTP) and phylogenetic analysis in Fig. 1. Column “clade assignation” refers to the results from the phylogenetic analysis and showed in Fig. 1.

GenBank acc. number	Strain	Origin	Original species assignation	Putative species assignation	Clade
KF717874	SAP1335	Ecuador	*Saprolegnia parasitica*	outgroup	outgroup
MH269708	M6_1	Finland	*Aphanomyces fennicus*	*Aphanomyces fennicus*	Clade 1A
MH269709	M6_2	Finland	*Aphanomyces fennicus*	*Aphanomyces fennicus*	Clade 1A
MH269710	M7_3	Finland	*Aphanomyces fennicus*	*Aphanomyces fennicus*	Clade 1A
AB533285	NJM 0705	Japan	*Aphanomyces sp*	*Aphanomyces* sp. 1	Clade 1A
AB533286	NJM 0708	Japan	*Aphanomyces sp*	*Aphanomyces* sp. 1	Clade 1A
AB533287	NJM 0706	Japan	*Aphanomyces sp*	*Aphanomyces* sp. 1	Clade 1A
AB533288	NJM 0707	Japan	*Aphanomyces sp*	*Aphanomyces* sp. 1	Clade 1A
EU443838	n/a	UK	*Aphanomyces frigidophilus*	*Aphanomyces frigidophilus*	Clade 1A
FM999233	SAP263	Spain	*Aphanomyces frigidophilus*	*Aphanomyces frigidophilus*	Clade 1A
FM992370	SAP472	Spain	*Aphanomyces frigidophilus*	*Aphanomyces frigidophilus*	Clade 1A
JX087973	WLL-2012 isolate 162	Canada	*Aphanomyces sp*	*Aphanomyces frigidophilus*	Clade 1A
JX087974	WLL-2012 isolate 167	Canada	*Aphanomyces sp*	*Aphanomyces frigidophilus*	Clade 1A
JX087975	WLL-2012 isolate 268	Canada	*Aphanomyces sp*	*Aphanomyces frigidophilus*	Clade 1A
JX087976	WLL-2012 isolate 269	Canada	*Aphanomyces sp*	*Aphanomyces frigidophilus*	Clade 1A
AY647192	n/a	Japan	*Aphanomyces frigidophilus*	*Aphanomyces frigidophilus*	Clade 1A
FM999229	SAP307	USA	*Aphanomyces invadans*	*Aphanomyces invadans*	Clade 1A
FM999231	SAP309	USA	*Aphanomyces invadans*	*Aphanomyces invadans*	Clade 1A
EU422990	NJM 9701	n/a	*Aphanomyces invadans*	*Aphanomyces invadans*	Clade 1A
AY455773	NJM 9801	Philippines	*Aphanomyces piscida*	*Aphanomyces invadans*	Clade 1A
MN814020	S15-19	India	*Aphanomyces invadans*	*Aphanomyces invadans*	Clade 1A
MK072633	J133	Bangladesh	*Aphanomyces invadans*	*Aphanomyces invadans*	Clade 1A
MK072635	Jrui	Bangladesh	*Aphanomyces invadans*	*Aphanomyces invadans*	Clade 1A
MK072636	Jkoi	Bangladesh	*Aphanomyces invadans*	*Aphanomyces invadans*	Clade 1A
MK072627	M87	Bangladesh	*Aphanomyces invadans*	*Aphanomyces invadans*	Clade 1A
MK072628	M106	Bangladesh	*Aphanomyces invadans*	*Aphanomyces invadans*	Clade 1A
MK072629	M223	Bangladesh	*Aphanomyces invadans*	*Aphanomyces invadans*	Clade 1A
MK072630	Mshol	Bangladesh	*Aphanomyces invadans*	*Aphanomyces invadans*	Clade 1A
MK072631	J78	Bangladesh	*Aphanomyces invadans*	*Aphanomyces invadans*	Clade 1A
AY283640	NJM 2024	Thailand	*Aphanomyces piscida*	*Aphanomyces invadans*	Clade 1A
AY283641	NJM 0003	Japan	*Aphanomyces piscida*	*Aphanomyces invadans*	Clade 1A
AY283642	RF6	Thailand	*Aphanomyces invadans*	*Aphanomyces invadans*	Clade 1A
AY283643	NJM 9803	Japan	*Aphanomyces piscida*	*Aphanomyces invadans*	Clade 1A
AY283644	NJM 0006	USA	*Aphanomyces piscida*	*Aphanomyces invadans*	Clade 1A
AY283645	NJM 0002	Japan	*Aphanomyces piscida*	*Aphanomyces invadans*	Clade 1A
FM999230	SAP308	USA	*Aphanomyces invadans*	*Aphanomyces invadans*	Clade 1A
FJ794883	APH1_SAN01 clone 1	n/a	*Aphanomyces sp*	*Aphanomyces* sp. 2	Clade 1A
FJ794884	APH1_SAN01 clone 2	n/a	*Aphanomyces sp*	*Aphanomyces* sp. 2	Clade 1A
FJ794885	APH1_SAN01 clone 3	n/a	*Aphanomyces sp*	*Aphanomyces* sp. 2	Clade 1A
FJ794886	APH1_SAN01 clone 4	n/a	*Aphanomyces sp*	*Aphanomyces* sp. 2	Clade 1A
FJ794887	APH1_SAN01 clone 5	n/a	*Aphanomyces sp*	*Aphanomyces* sp. 2	Clade 1A
FJ794888	APH1_SAN01 clone 6	n/a	*Aphanomyces sp*	*Aphanomyces* sp. 2	Clade 1A
FJ794889	APH1_VIR01 clone 1	n/a	*Aphanomyces sp*	*Aphanomyces* sp. 2	Clade 1A
FJ794890	APH1_VIR01 clone 2	n/a	*Aphanomyces sp*	*Aphanomyces* sp. 2	Clade 1A
FJ794891	APH1_VIR01 clone 3	n/a	*Aphanomyces sp*	*Aphanomyces* sp. 2	Clade 1A
FJ794892	APH1_VIR01 clone 4	n/a	*Aphanomyces sp*	*Aphanomyces* sp. 2	Clade 1A
FJ794893	APH1_VIR01 clone 5	n/a	*Aphanomyces sp*	*Aphanomyces* sp. 2	Clade 1A
MH517688	UEF_T2JN1	USA	*Aphanomyces sp*	*Aphanomyces* sp. 2	Clade 1A
MH517685	UEF_T2S2	USA	*Aphanomyces sp*	*Aphanomyces* sp. 2	Clade 1A
MH517686	UEF_T2S1	USA	*Aphanomyces sp*	*Aphanomyces* sp. 2	Clade 1A
MH517687	UEF_T2JN2	USA	*Aphanomyces sp*	*Aphanomyces* sp. 2	Clade 1A
MH517684	UEF_T2S3	USA	*Aphanomyces sp*	*Aphanomyces* sp. 2	Clade 1A
AB510348	NJM0801	Japan	*Aphanomyces salsuginosus*	*Aphanomyces salsuginosus*	Clade 1A
AB510349	NJM0802	Japan	*Aphanomyces salsuginosus*	*Aphanomyces salsuginosus*	Clade 1A
AB510350	NJM0803	Japan	*Aphanomyces salsuginosus*	*Aphanomyces salsuginosus*	Clade 1A
AB510351	NJM0804	Japan	*Aphanomyces salsuginosus*	*Aphanomyces salsuginosus*	Clade 1A
AB510352	NJM0805	Japan	*Aphanomyces salsuginosus*	*Aphanomyces salsuginosus*	Clade 1A
AB533297	NJM 0911	Japan	*Aphanomyces sp*	*Aphanomyces salsuginosus*	Clade 1A
AB533298	NJM 0913	Japan	*Aphanomyces sp*	*Aphanomyces salsuginosus*	Clade 1A
AB533299	NJM 0918	Japan	*Aphanomyces sp*	*Aphanomyces salsuginosus*	Clade 1A
AB533300	NJM 0919	Japan	*Aphanomyces sp*	*Aphanomyces salsuginosus*	Clade 1A
AB533301	NJM 0903	Japan	*Aphanomyces sp*	*Aphanomyces salsuginosus*	Clade 1A
AB533303	NJM 0905	Japan	*Aphanomyces sp*	*Aphanomyces salsuginosus*	Clade 1A
AB533305	NJM 0907	Japan	*Aphanomyces sp*	*Aphanomyces salsuginosus*	Clade 1A
AY283647	IA1651	Japan	*Aphanomyces stellatus*	*Aphanomyces stellatus2*	Clade 1A
AY283648	IA1833	Japan	*Aphanomyces laevis*	*Aphanomyces stellatus3*	Clade 1A
AY455774	IA0029	Japan	*Aphanomyces stellatus*	*Aphanomyces stellatus3*	Clade 1A
AM947029	CBS568,67 = VI03561	n/a	*Aphanomyces stellatus*	*Aphanomyces stellatus3*	Clade 1A
KP006462	CCIBt 3994	Brazil?	*Aphanomyces stellatus*	*Aphanomyces stellatus3*	Clade 1A
MK513784	CCIBt 4305	Brazil	*Aphanomyces stellatus*	*Aphanomyces stellatus3*	Clade 1A
KY989960	CNUaq	South Korea	*Aphanomyces sp*	*Aphanomyces brasiliensis*	Clade 1B
EF175576	MCA 3058	n/a	*Aphanomyces sp*	*Aphanomyces brasiliensis*	Clade 1B
MK513785	CCIBt 4359	Brazil	*Aphanomyces brasiliensis*	*Aphanomyces brasiliensis*	Clade 1B
MK513786	CPZ14	Brazil	*Aphanomyces brasiliensis*	*Aphanomyces brasiliensis*	Clade 1B
AB531971	NJM 0719	Japan	*Aphanomyces sinensis*	*Aphanomyces sinensis*	Clade 1B
AB531972	NJM 0817	Japan	*Aphanomyces sinensis*	*Aphanomyces sinensis*	Clade 1B
AB531973	NJM 0818	Japan	*Aphanomyces sinensis*	*Aphanomyces sinensis*	Clade 1B
AB531974	NJM 0901	Japan	*Aphanomyces sinensis*	*Aphanomyces sinensis*	Clade 1B
AB531975	NJM 0902	Japan	*Aphanomyces sinensis*	*Aphanomyces sinensis*	Clade 1B
JQ070116	ATCC MYA-4825	n/a	*Aphanomyces sinensis*	*Aphanomyces sinensis*	Clade 1B
AB533282	NJM 0703	n/a	*Aphanomyces sp*	*Aphanomyces* sp. 6	Clade 1B
AB533283	NJM 0704	n/a	*Aphanomyces sp*	*Aphanomyces* sp. 6	Clade 1B
AB533284	NJM 0714	n/a	*Aphanomyces sp*	*Aphanomyces* sp. 6	Clade 1B
FM999222	SAP369	Canada	*Aphanomyces cladogamus*	*Aphanomyces cladogamus*	Clade 2
FM999228	SAP355	USA	*Aphanomyces cladogamus*	*Aphanomyces cladogamus*	Clade 2
AY353912	Strain 4	n/a	*Aphanomyces cladogamus*	*Aphanomyces cladogamus*	Clade 2
AY353913	Strain 7	n/a	*Aphanomyces cladogamus*	*Aphanomyces cladogamus*	Clade 2
AY353914	Strain 9	n/a	*Aphanomyces cladogamus*	*Aphanomyces cladogamus*	Clade 2
AY353915	Strain 14	n/a	*Aphanomyces cladogamus*	*Aphanomyces cladogamus*	Clade 2
AY353916	Strain 15	n/a	*Aphanomyces cladogamus*	*Aphanomyces cladogamus*	Clade 2
AY353917	Strain 91	n/a	*Aphanomyces cladogamus*	*Aphanomyces cladogamus*	Clade 2
AY353918	Strain 87	n/a	*Aphanomyces cladogamus*	*Aphanomyces cladogamus*	Clade 2
AY353919	Strain 96	n/a	*Aphanomyces cladogamus*	*Aphanomyces cladogamus*	Clade 2
AY353920	CBS 128.29	n/a	*Aphanomyces cladogamus*	*Aphanomyces cladogamus*	Clade 2
HQ643112	CBS 69,079	Canada	*Aphanomyces cladogamus*	*Aphanomyces cladogamus*	Clade 2
HQ643113	CBS 10,829	n/a	*Aphanomyces cladogamus*	*Aphanomyces cladogamus*	Clade 2
HQ643114	BR 693	Canada	*Aphanomyces cladogamus*	*Aphanomyces cladogamus*	Clade 2
GQ267547	TAOR- 2010 isolate UWA042-13	Australia	*Aphanomyces trifolii*	*Aphanomyces trifolii*	Clade 2
GQ267548	TAOR- 2010 isolate UWA009-1	Australia	*Aphanomyces trifolii*	*Aphanomyces trifolii*	Clade 2
GQ267549	TAOR- 2010 isolate UWA007-5	Australia	*Aphanomyces trifolii*	*Aphanomyces trifolii*	Clade 2
GQ267550	TAOR- 2010 isolate UWA007-3	Australia	*Aphanomyces trifolii*	*Aphanomyces trifolii*	Clade 2
GQ267551	TAOR- 2010 isolate UWA010-6	Australia	*Aphanomyces trifolii*	*Aphanomyces trifolii*	Clade 2
GQ267552	TAOR- 2010 isolate UWA041-6	Australia	*Aphanomyces trifolii*	*Aphanomyces trifolii*	Clade 2
GQ267553	TAOR- 2010 isolate UWA040-8	Australia	*Aphanomyces trifolii*	*Aphanomyces trifolii*	Clade 2
GQ267554	TAOR- 2010 isolate UWA040-5	Australia	*Aphanomyces trifolii*	*Aphanomyces trifolii*	Clade 2
MK513783	CCIBt4409	Brazil	*Aphanomyces raphani*	*Aphanomyces raphani*	Clade 2
FM999208	SAP344	USA	*Aphanomyces cochlioides*	*Aphanomyces cochlioides*	Clade 2
FM999209	SAP345	USA	*Aphanomyces cochlioides*	*Aphanomyces cochlioides*	Clade 2
FM999210	SAP346	USA	*Aphanomyces cochlioides*	*Aphanomyces cochlioides*	Clade 2
FM999211	SAP347	USA	*Aphanomyces cochlioides*	*Aphanomyces cochlioides*	Clade 2
FM999212	SAP348	USA	*Aphanomyces cochlioides*	*Aphanomyces cochlioides*	Clade 2
FM999213	SAP349	USA	*Aphanomyces cochlioides*	*Aphanomyces cochlioides*	Clade 2
FM999215	SAP351	USA	*Aphanomyces cochlioides*	*Aphanomyces cochlioides*	Clade 2
FM999217	SAP359	USA	*Aphanomyces cochlioides*	*Aphanomyces cochlioides*	Clade 2
FM999218	SAP360	USA	*Aphanomyces cochlioides*	*Aphanomyces cochlioides*	Clade 2
FM999219	SAP361	USA	*Aphanomyces cochlioides*	*Aphanomyces cochlioides*	Clade 2
FM999220	SAP362	USA	*Aphanomyces cochlioides*	*Aphanomyces cochlioides*	Clade 2
FM999221	SAP365	USA	*Aphanomyces cochlioides*	*Aphanomyces cochlioides*	Clade 2
FM999222	SAP369	USA	*Aphanomyces cladogamus*	*Aphanomyces cladogamus*	Clade 2
FM999223	SAP371	USA	*Aphanomyces cochlioides*	*Aphanomyces cochlioides*	Clade 2
FM999224	SAP372	USA	*Aphanomyces cochlioides*	*Aphanomyces cochlioides*	Clade 2
FM999216	SAP358	USA	*Aphanomyces cochlioides*	*Aphanomyces cochlioides*	Clade 2
AY353911	Strain 94	n/a	*Aphanomyces cochlioides*	*Aphanomyces cochlioides*	Clade 2
HQ643115	CBS47771	n/a	*Aphanomyces cochlioides*	*Aphanomyces cochlioides*	Clade 2
KJ744312	Strain PM1	Germany	*Aphanomyces cochlioides*	*Aphanomyces cochlioides*	Clade 2
KJ744369	Strain 2012–003	Germany	*Aphanomyces cochlioides*	*Aphanomyces cochlioides*	Clade 2
KJ744370	Strain 2012–003	Germany	*Aphanomyces cochlioides*	*Aphanomyces cochlioides*	Clade 2
FM999214	SAP350	USA	*Aphanomyces cochlioides*	*Aphanomyces cochlioides*	Clade 2
AY353921	65	n/a	*Aphanomyces sp*	*Aphanomyces* sp. 7	Clade 2
AY647190	MAFF305549	Japan	*Aphanomyces euteiches*	*Aphanomyces euteiches*	Clade 2
KY593270	Isolate A2	Canada	*Aphanomyces euteiches*	*Aphanomyces euteiches*	Clade 2
KM486067	Isolate 206	Canada	*Aphanomyces euteiches*	*Aphanomyces euteiches*	Clade 2
KT896668	SD_FP1	USA	*Aphanomyces euteiches*	*Aphanomyces euteiches*	Clade 2
KT896667	SD_LN1	USA	*Aphanomyces euteiches*	*Aphanomyces euteiches*	Clade 2
KM486065	Isolate 315	Canada	*Aphanomyces euteiches*	*Aphanomyces euteiches*	Clade 2
KM486066	Isolate 1309	Canada	*Aphanomyces euteiches*	*Aphanomyces euteiches*	Clade 2
FM999225	SAP357	USA	*Aphanomyces euteiches*	*Aphanomyces euteiches*	Clade 2
KT285705	Isolate 55	n/a	*Aphanomyces euteiches*	*Aphanomyces euteiches*	Clade 2
AY683887	ATCC 201,684	n/a	*Aphanomyces euteiches*	*Aphanomyces euteiches*	Clade 2
AY283646	IA0475	Japan	*Aphanomyces laevis*	*Aphanomyces euteiches*	Clade 2
FM999226	SAP368	Norway	*Aphanomyces euteiches*	*Aphanomyces euteiches*	Clade 2
JX418020	SAP1479	Denmark	*Aphanomyces euteiches*	*Aphanomyces euteiches*	Clade 2
HQ643119	CBS15473	Norway	*Aphanomyces euteiches*	*Aphanomyces euteiches*	Clade 2
AY353901	Strain R	n/a	*Aphanomyces euteiches*	*Aphanomyces euteiches*	Clade 2
AY353902	Strain 5	n/a	*Aphanomyces euteiches*	*Aphanomyces euteiches*	Clade 2
AY353903	Strain 63	n/a	*Aphanomyces euteiches*	*Aphanomyces euteiches*	Clade 2
AY353904	Strain 57	n/a	*Aphanomyces euteiches*	*Aphanomyces euteiches*	Clade 2
AY353905	Strain 11	n/a	*Aphanomyces euteiches*	*Aphanomyces euteiches*	Clade 2
AY353906	Strain 109	n/a	*Aphanomyces euteiches*	*Aphanomyces euteiches*	Clade 2
AY353907	Strain MF1 race1	n/a	*Aphanomyces euteiches*	*Aphanomyces euteiches*	Clade 2
AY353908	MHA41 race2	n/a	*Aphanomyces euteiches*	*Aphanomyces euteiches*	Clade 2
AY353909	Strain 110	n/a	*Aphanomyces euteiches*	*Aphanomyces euteiches*	Clade 2
AY353910	ATCC 46,688	n/a	*Aphanomyces euteiches f.sp. phaseoli*	*Aphanomyces euteiches*	Clade 2
HQ643116	CBS15773	Norway	*Aphanomyces euteiches*	*Aphanomyces euteiches*	Clade 2
HQ643117	CBS15673	Norway	*Aphanomyces euteiches*	*Aphanomyces euteiches*	Clade 2
HQ643118	CBS15573	Norway	*Aphanomyces euteiches*	*Aphanomyces euteiches*	Clade 2
HQ643120	BR694	Canada	*Aphanomyces euteiches*	*Aphanomyces euteiches*	Clade 2
FM999227	SAP356	Japan	*Aphanomyces iridis*	*Aphanomyces iridis*	Clade 2
HQ643121	CBS52487	??	*Aphanomyces iridis*	*Aphanomyces iridis*	Clade 2
KU209669	MICO 5–28 1	USA	*Aphanomyces cladogamus*	*Aphanomyces* sp. 7	Clade 2
MN365601	ACITS64	n/a	*Aphanomyces cladogamus*	*Aphanomyces* sp. 7	Clade 2
MK326481	MICO 5–28 1	n/a	*Aphanomyces cladogamus*	*Aphanomyces* sp. 7	Clade 2
KU209452	KSSO 5–30	USA	*Aphanomyces cochlioides*	*Aphanomyces* sp. 7	Clade 2
DQ889219	AW-1	n/a	*Aphanomyces sp*	*Aphanomyces* sp. 8	Clade 2
DQ861921	AW-1	n/a	*Aphanomyces sp*	*Aphanomyces* sp. 8	Clade 2
HQ643402	CBS52387	Japan	*Plectospira myriandra*	*Plectospira myriandra*	Clade 3
KX084705	GHJ01	Brazil	*Plectospira gemmifera*	*Plectospira* sp. 1	Clade 3
KX353798	CCIBt 3992	Brazil	*Plectospira myriandra*	*Plectospira gemmifera*	Clade 3
KX353797	CCIBt 3372	Brazil	*Plectospira myriandra*	*Plectospira gemmifera*	Clade 3
KR063220	CCIBt 3992	Brazil	*Plectospira myriandra*	*Plectospira gemmifera*	Clade 3
KT935288	CCIBt3372	Brazil	*Plectospira myriandra*	*Plectospira gemmifera*	Clade 3
FM999237	SAP366	Germany	*Aphanomyces laevis*	*Aphanomyces repetans*	Clade 1B
FM999234	SAP54	Spain	*Aphanomyces repetans*	*Aphanomyces repetans*	Clade 1B
KF386652	SAP761	Czech Republic	*Aphanomyces sp*	*Aphanomyces repetans*	Clade 1B
KF386651	SAP760	Czech Republic	*Aphanomyces sp*	*Aphanomyces repetans*	Clade 1B
AY683892	Fa	n/a	*Aphanomyces repetans*	*Aphanomyces repetans*	Clade 1B
KF386653	SAP667	Czech Republic	*Aphanomyces sp*	*Aphanomyces repetans*	Clade 1B
KP663634	263_AH	n/a	*Aphanomyces scaber*	*Aphanomyces repetans*	Clade 1B
KP663635	264_AH	n/a	*Aphanomyces scaber*	*Aphanomyces repetans*	Clade 1B
KF386649	SAP678	Czech Republic	*Aphanomyces sp*	*Aphanomyces repetans*	Clade 1B
KF386650	SAP659	Czech Republic	*Aphanomyces sp*	*Aphanomyces repetans*	Clade 1B
HQ643122	CBS47871	Germany	*Aphanomyces laevis*	*Aphanomyces repetans*	Clade 1B
AY683889	An	n/a	*Aphanomyces repetans*	*Aphanomyces repetans*	Clade 1B
GU182321	13,686/TE	Italy	*Aphanomyces repetans*	*Aphanomyces repetans*	Clade 1B
FM999235	SAP60	Spain	*Aphanomyces repetans*	*Aphanomyces repetans*	Clade 1B
AY683897	Se	n/a	*Aphanomyces repetans*	*Aphanomyces* sp. 3	Clade 1B
HQ111466	CBS 126,887	n/a	*Aphanomyces sp*	*Aphanomyces* sp. 3	Clade 1B
HQ111457	CBS 126,886	n/a	*Aphanomyces sp*	*Aphanomyces* sp. 3	Clade 1B
AY676019	F-1293	France	*Aphanomyces sp*	*Aphanomyces* sp. 3	Clade 1B
AB533289	NJM 0908	Japan	*Aphanomyces sp*	*Aphanomyces* sp. 5	Clade 1B
AB533290	NJM 0909	Japan	*Aphanomyces sp*	*Aphanomyces* sp. 5	Clade 1B
AB533291	NJM 0910	Japan	*Aphanomyces sp*	*Aphanomyces* sp. 5	Clade 1B
AB533292	NJM 0912	Japan	*Aphanomyces sp*	*Aphanomyces* sp. 5	Clade 1B
AB533293	NJM 0914	Japan	*Aphanomyces sp*	*Aphanomyces* sp. 5	Clade 1B
AB533294	NJM 0915	Japan	*Aphanomyces sp*	*Aphanomyces* sp. 5	Clade 1B
AB533295	NJM 0916	Japan	*Aphanomyces sp*	*Aphanomyces* sp. 5	Clade 1B
AB533296	NJM 0917	Japan	*Aphanomyces sp*	*Aphanomyces* sp. 5	Clade 1B
AY647191	MAFF305548	Japan	*Aphanomyces cochlioides*	*Aphanomyces* sp. 5	Clade 1B
KP006463	CCIBt 4070	Brazil	*Aphanomyces laevis*	*Aphanomyces* sp. 5	Clade 1B
AM947028	CBS 105.52	n/a	*Aphanomyces laevis*	*Aphanomyces laevis*	Clade 1B
AY310497	CBS 107.52	n/a	*Aphanomyces laevis*	*Aphanomyces laevis*	Clade 1B
AY683885	CBS 465.64	n/a	*Aphanomyces laevis*	*Aphanomyces laevis*	Clade 1B
HQ643123	CBS 58,385	USA	*Aphanomyces laevis*	*Aphanomyces laevis*	Clade 1B
KR063218	CCIBt 3987	Brazil	*Aphanomyces helicoides*	*Aphanomyces* sp. 4	Clade 1B
MK513782	CPZ100	Brazil	*Aphanomyces helicoides*	*Aphanomyces* sp. 4	Clade 1B
MK513781	CCIBt4331	Brazil	*Aphanomyces helicoides*	*Aphanomyces* sp. 4	Clade 1B
FM999238	SAP367	Russia	*Aphanomyces helicoides*	*Aphanomyces helicoides*	Clade 1B
AY310496	CBS 210.82	n/a	*Aphanomyces helicoides*	*Aphanomyces helicoides*	Clade 1B
KT213554	CCIBt 3986	Brazil	*Phragmosporangium uniseratum*	*Phragmosporangium uniseratum*	Clade 1A
KT935286	CCIBt 4107	Brazil	*Phragmosporangium uniseratum*	*Phragmosporangium uniseratum*	Clade 1A
KT935287	CCIBt 2332	Brazil	*Phragmosporangium uniseratum*	*Phragmosporangium uniseratum*	Clade 1A
PX602887	CCRJB_6	Spain	*Aphanomyces astaci*	*Aphanomyces astaci*	Clade 1A
PX602888	CCRJB_14	Spain	*Aphanomyces astaci*	*Aphanomyces astaci*	Clade 1A
PX602889	CCRJB_26	Spain	*Aphanomyces astaci*	*Aphanomyces astaci*	Clade 1A
PX602890	CCRJB_39	Spain	*Aphanomyces astaci*	*Aphanomyces astaci*	Clade 1A
PX602891	CCRJB_48	Spain	*Aphanomyces astaci*	*Aphanomyces astaci*	Clade 1A
PX602892	CCRJB_63	Spain	*Aphanomyces astaci*	*Aphanomyces astaci*	Clade 1A
PX602893	CCRJB_37	Finland	*Aphanomyces astaci*	*Aphanomyces astaci*	Clade 1A
PX602894	CCRJB_64	Finland	*Aphanomyces astaci*	*Aphanomyces astaci*	Clade 1A
PX602895	CCRJB_70	Spain	*Aphanomyces astaci*	*Aphanomyces astaci*	Clade 1A
PX602896	CCRJB_74	Finland	*Aphanomyces astaci*	*Aphanomyces astaci*	Clade 1A
PX602897	CCRJB_60	Spain	*Aphanomyces astaci*	*Aphanomyces astaci*	Clade 1A
PX602898	CCRJB_53	Spain	*Aphanomyces astaci*	*Aphanomyces astaci*	Clade 1A
PX602899	CCRJB_31	Spain	*Aphanomyces astaci*	*Aphanomyces astaci*	Clade 1A
PX602900	CCRJB_50	Spain	*Aphanomyces astaci*	*Aphanomyces astaci*	Clade 1A
PX602901	CCRJB_62	Spain	*Aphanomyces astaci*	*Aphanomyces astaci*	Clade 1A
PX602902	CCRJB_79	Finland	*Aphanomyces astaci*	*Aphanomyces astaci*	Clade 1A
PX602903	CCRJB_49	Spain	*Aphanomyces astaci*	*Aphanomyces astaci*	Clade 1A
PX602904	CCRJB_55	Chile	*Aphanomyces astaci*	*Aphanomyces astaci*	Clade 1A
PX602905	CCRJB_66	Finland	*Aphanomyces astaci*	*Aphanomyces astaci*	Clade 1A
PX602906	CCRJB_10	Chile	*Aphanomyces frigidophilus*	*Aphanomyces frigidophilus*	Clade 1A
PX602907	CCRJB_69	Jaoan	*Aphanomyces salsuginosus*	*Aphanomyces salsuginosus*	Clade 1A
PX602909	CCRJB_61	Japan	*Aphanomyces salsuginosus*	*Aphanomyces salsuginosus*	Clade 1A
PX602910	CCRJB_47	Italy	*Aphanomyces stellatus*	*Aphanomyces stellatus*	Clade 1A
PX602911	CCRJB_17	Finland	*Aphanomyces stellatus*	*Aphanomyces stellatus*	Clade 1A
PX602912	CCRJB_71	Spain	*Aphanomyces stellatus*	*Aphanomyces stellatus*	Clade 1A
PX602913	CCRJB_72	Spain	*Aphanomyces stellatus*	*Aphanomyces stellatus*	Clade 1A
PX602908	CCRJB_59	Chile	*Aphanomyces stellatus*	*Aphanomyces stellatus*	Clade 1A
PX602914	CCRJB_1	Costa Rica	*Aphanomyces sp*	*Aphanomyces brasiliensis*	Clade 1B
PX602915	CCRJB_30	Ecuador	*Aphanomyces sp*	*Aphanomyces brasiliensis*	Clade 1B
PX602916	CCRJB_51	Ecuador	*Aphanomyces sp*	*Aphanomyces brasiliensis*	Clade 1B
PX602917	CCRJB_22	Japan	*Aphanomyces sinensis*	*Aphanomyces sinensis*	Clade 1B
PX602918	CCRJB_8	USA	*Aphanomyces cochlioides*	*Aphanomyces cochlioides*	Clade 2
PX602919	CCRJB_13	USA	*Aphanomyces cochlioides*	*Aphanomyces cochlioides*	Clade 2
PX602920	CCRJB_25	USA	*Aphanomyces cochlioides*	*Aphanomyces cochlioides*	Clade 2
PX602921	CCRJB_32	USA	*Aphanomyces cochlioides*	*Aphanomyces cochlioides*	Clade 2
PX602922	CCRJB_38	USA	*Aphanomyces cochlioides*	*Aphanomyces cochlioides*	Clade 2
PX602923	CCRJB_43	USA	*Aphanomyces cochlioides*	*Aphanomyces cochlioides*	Clade 2
PX602924	CCRJB_40	Norway	*Aphanomyces euteiches*	*Aphanomyces euteiches*	Clade 2
PX602925	CCRJB_27	Australia	*Plectospira myriandra*	*Plectospira myriandra*	Clade 3
PX602926	CCRJB_19	Spain	*Aphanomyces laevis-repetans*	*Aphanomyces repetans*	Clade 1B
PX602930	CCRJB_20	Spain	*Aphanomyces laevis-repetans*	*Aphanomyces repetans*	Clade 1B
PX602929	CCRJB_45	Finland	*Aphanomyces laevis-repetans*	*Aphanomyces repetans*	Clade 1B
PX602928	CCRJB_46	Spain	*Aphanomyces laevis-repetans*	*Aphanomyces repetans*	Clade 1B
PX602927	CCRJB_34	Ecuador	*Aphanomyces laevis-repetans*	*Aphanomyces repetans*	Clade 1B
PX602931	CCRJB_52	Japan	*Aphanomyces sp*	*Aphanomyces* sp. 5	Clade 1B
PX602932	CCRJB_2	Finland	*Aphanomyces laevis-repetans*	*Aphanomyces laevis*	Clade 1B
PX602933	CCRJB_23	Finland	*Aphanomyces laevis-repetans*	*Aphanomyces laevis*	Clade 1B
PX602934	CCRJB_54	Finland	*Aphanomyces laevis-repetans*	*Aphanomyces laevis*	Clade 1B
PX602935	CCRJB_41	Finland	*Aphanomyces laevis-repetans*	*Aphanomyces laevis*	Clade 1B
PX602936	CCRJB_18	Finland	*Aphanomyces laevis-repetans*	*Aphanomyces laevis*	Clade 1B
PX602937	CCRJB_16	Finland	*Aphanomyces laevis-repetans*	*Aphanomyces laevis*	Clade 1B

We conducted DNA amplification targeting the nuclear ribosomal Internal Transcriber Spacer (nrITS) region using the primer pair ITS4 and ITS5^21^. The PCR mix contained 15.2 μL miliQ H20, 2.5 μL10x Buffer, 1.6 μL 2.5 mM dNTP, 1.4 μL 50 mM MgCl2, 1 μL 1 mg/mL BSA, 1 μL 10 mM of each primer, 0.3 μL 5u/mL BIOTAQ DNA Polymerase and 1 μL of DNA template. The PCR reactions were conducted using the following conditions: 95ºC, 2 min, 35x (95ºC, 1 min; 52ºC, 25 s; 72ºC, 45 s) and 72ºC 10 min in a Mastercycler X50S thermocycler (Eppendorf). Each round of PCR contained a positive and a negative control. The positive control consisted on 1 μL DNA from *A. astaci* CCRJB_70 strain and the negative control contained 1 μL nuclease-free water. PCR products were checked on a 1% TAE-buffered agarose gel stained with SYBR Safe (Thermo Fisher Scientific, Waltham, MA, USA). The amplified products were first purified using a QIAquick PCR Purification Kit (QIAGEN, Chatsworth, CA), and subsequently Sanger sequenced using a 3730xl DNA automated sequencer (Applied Biosystems) at the external server Macrogen (Madrid, Spain) using the same primers as for the amplification.

### Molecular dataset

We downloaded all available sequences from *Aphanomyces* from GenBank (NCBI database; Table 1). Misidentified accessions and those of low quality (high number of ambiguous bases) were removed from the dataset. We also included the newly generated sequences from the RJB-CSIC culture collection in the matrix. The sequences were visualized, edited and aligned using the MAFFT algorithm^23^ with default parameters, as implemented in Geneious 11.0^22^.

### Molecular species delimitation

Previous to the species delimitation analyses, we collapsed the sequence matrix into haplotypes using the web server FaBox 1.61 [^24^ https://birc.au.dk/~palle/php/fabox/]. Four species delimitation analyses were conducted independently using the following methods: (i) Automatic Barcode Gap Delimitation (ABGD^25^), (ii) Assemble Species by Automatic Partitioning (ASAP^26^), (iii) Poisson Tree Processes (bPTP^27^), and (iv) General Mixed Yule Coalescence (GMYC^28,29^). The ABGD and ASAP are distance-based approaches, whereas the bPTP and GMYC analyses are phylogeny-based. Thus, both ABGD and ASAP search for a barcode gap in the distribution of pairwise sequence distances, and use it as a threshold to delimit candidate species or molecular operational taxonomic units (MOTUs). The main difference between both approaches is that ABGD requires prior biological insight into intraspecific diversity^25^, contrary to ASAP, which also provides a score to rank partitioning schemes^26^. In contrast, the phylogeny-based methods require the previous inference of a phylogenetic hypothesis for the target group. The bPTP uses the number of substitutions to estimate branching processes using a Poisson distribution^27^. Instead, the GMYC model requires an ultrametric tree as an input to infer the transition threshold between branching events caused by speciation and corresponding to a Yule process, and those occurring at the intraspecific level, modelled as a coalescence process^28^. The GMYC method allows the approach to be implemented using a single or multiple threholds^28,29^.

The ABGD, ASAP and bPTP analyses were performed on their respective online implementations (https://bioinfo.mnhn.fr/abi/public/abgd/abgdweb.html for ABGD, https://bioinfo.mnhn.fr/abi/public/asap/asapweb.html for ASAP and https://species.h-its.org/ptp/ for bPTP). The genetic distance matrices used to perform the ABGD and ASAP algorithms were calculated in the nrITS alignment using the Kimura substitution model (K80). ABGD was implemented using default parameters but for X = 1. For the bPTP analysis, we used a Bayesian Inference (BI) rooted tree as input, in which the outgroup had been previously removed. The BI tree was inferred in the MrBayes v.3.2.6 software^30^ using the TPM2uf + G substitution model after selection among 20 substitution schemes in jModelTest v.2.1.10^31^, and according to following parameters: 10 M generation using the MCMC method, two runs (4 chains each of them) and a burn-in of 25% trees. The ultrametric tree required to implement the GMYC algorithm was inferred in BEAST v1.10.4^32^ using the nrITS alignment collapsed into haplotypes. We selected the GTR + G as substitution model after selection among 11 substitution schemes in jModelTest v.2.1.10^31^ using the Bayesian Information Criterion. An UPGMA tree was selected as starting tree. We used a strict clock prior^33^ and a fixed arbitrary substitution rate of 1. We chose a speciation Yule process as tree prior. We ran the generated xml file in BEAST for 20,000,000 generations sampling every 5,000 generations. We used TreeAnnotator v1.10.4^32^ to construct a maximum credibility ultrametric tree from the tree file removing 25% of all trees sampled as burn-in. Finally, we ran the GMYC analyses with the generated ultrametric tree using the gmyc function of the splits package^34^ in R v.3.4.1, testing the both single and multiple threshold versions. Figure editing was performed using Adobe Illustrator CC 2019 23.0.

### Phylogenetic analysis

For the phylogenetic analyses, we used the previously generated nrITS matrix, including *Saprolegnia parasitica* (SAP1335 strain; GenBank accession: KF717874) as outgroup. We employed jModelTest v.2.1.10^31^ under the Bayesian Information Criterion to determine the best substitution model for the 261-sequence dataset, resulting in the TPM2uf + G model. Phylogenetic relationships among species were inferred using Bayesian inference (BI) and maximum-likelihood (ML). The BI analysis was performed in MrBayes v.3.2.6 software^30^ with 10 M generations sampling every 5,000-step using the MCMC method, two runs (4 chains each of them) and a burn-in of 25% trees. The ML analysis was performed in RAxML v.8 program^35^ implemented in raxmlGUI v1.5b1^36^. It consisted of 1,000 independent searches and 1,000 rapid bootstraps to assign node confidence. We considered well supported nodes those with posterior probabilities (pp) ≥ 0.95 for the BI analysis and bootstrap (bs) ≥ 75 for the ML analysis. Resulting trees were visualized using FigTree v1.4.2^37^ and edited in Adobe Illustrator CC 2019 23.0.

### Host preference database

We compiled all information available on interactions between *Aphanomyces*, *Phragmosporangium* and *Plectospira* species and their hosts recording the following information: (i) oomycete species, (ii) host or substrate of the oomycete (to the species level, if available), (iii) host family, (iv) geographical origin, (v) GenBank sequence accession if available, and (vi) bibliographic reference. We defined an interaction between a pathogen and a host (a record in the matrix) as the species occurring and being characterized by morphologic analysis, isolation or sequencing from a known host or substrate. Samples obtained from substrate might still belong to species that could be host-specific, but have been simply isolated from substrate at some stages of the life cycle of the species. We first performed a review of the literature in Google Scholar using the key word “Aphanomyces” followed by the name of the species, e.g., “Aphanomyces” AND “invadans”, and the additional words “Host” AND “Range”. All the recovered publications were screened to evaluate the availability of required data for the database. Secondly, we searched for available accession numbers of the three target genera in GenBank, ensuring no duplicates between GenBank and literature records. However, only host records associated to a GenBank accession numbers could be named accordingly to the results of the MOTUs classifications of the present study.

### Interaction network analysis

Once the database was completed, we built two interaction matrices representing the compiled interactions between *Aphanomyces*, *Phragmosporangium* and *Plectospira* species and their hosts. The first matrix included information about interaction frequency, which was interpreted here as the number of records in our database in which a species was observed, isolated and/or sequenced from a particular host (weighted matrix). This approach is biased since it partially reflects the efforts and interests made in the literature towards certain particular taxa. However, the highest number of records of certain interactions may also reflect a prevalent specialization patterns among species. To contrast the patterns observed in the weighted matrix, we built a second matrix which contained only information about the presence or absence of the interaction (binary matrix). The interaction networks were represented graphically using the plotweb function from the R package bipartite^38^, and artwork was further edited in Adobe Illustrator CC 2019 23.0. We calculated several metrics to describe the architecture of the interaction networks: i) connectance, the ratio between the number of registered links and the total number of possible links, ii) nestedness, a pattern that appear when the more specialised species of a guild interact with a subset of the partners with which the more generalised species do, and iii) modularity, a measure of the extent to which interactions in the network occur between groups of species that interact mostly with each other but much less with other species in the network (modules) or not at all with other species in the network (compartments). To calculate connectance and nestedness, we used the networklevel function from the R package bipartite. For nestedness, we used the NODF index (Nestedness metric based on Overlap and Decreasing Fill)^39^ and its weighted version (wNODF)^40^. To calculate modularity, we used the function computemodules from the package bipartite which uses the Newman algorithm to calculate modularity for presence-absence matrices^41^, and the Beckett algorithm for the weighted matrix^42^. Since the algorithms calculate the likelihood of the modularity in an iterative process, we repeated the calculations 20 times and selected the run with the highest probability. All calculations were run during 107 steps using the DIRT-LPA algorithm which is less prone to get trap in local minima^38^. In addition, we calculated two specialization metrics for the weighted matrix: i) the network specialization parameter H_2_’, a measure of the degree of specialization of the entire network based on interactions frequencies, and ii) the species specialization parameter d’, the standardized specialization of each species in relation to the other species in the interaction network, measured using quantitative interactions based on the standardized Kullback-Leiber distance^43^. To calculate the H_2_’ parameter, we also used the networklevel function from the package bipartite. For the species specialization d’ values, we used the dfun function in the bipartite package^43^.

The significance of the obtained values for all network metrics except for connectance was calculated comparing them against null models. We generated 1000 random matrices from our weighted matrix using the vallnull function that implements the null model proposed by^44^ which constrains connectance and moderately constrains the marginal totals. For the binary matrix, we generated a null model of 1,000 random matrices using mgen function that implemented the algorithm proposed by^45^. We evaluated the significance of the networks parameters calculating z-scores as (X_observed_- µ_null_)/σ_null_, being X_observed_ the actual value of the parameter, µ_null_ the mean of the parameter for the matrices in the null model, and σ_null_ their standard deviation. We calculated p-values for the z-scores as the number of elements of the null model showing higher or fewer values than the observed value divided by the total number of elements in the null model.

## Results

### Molecular species delimitation

**Fig. 1:**
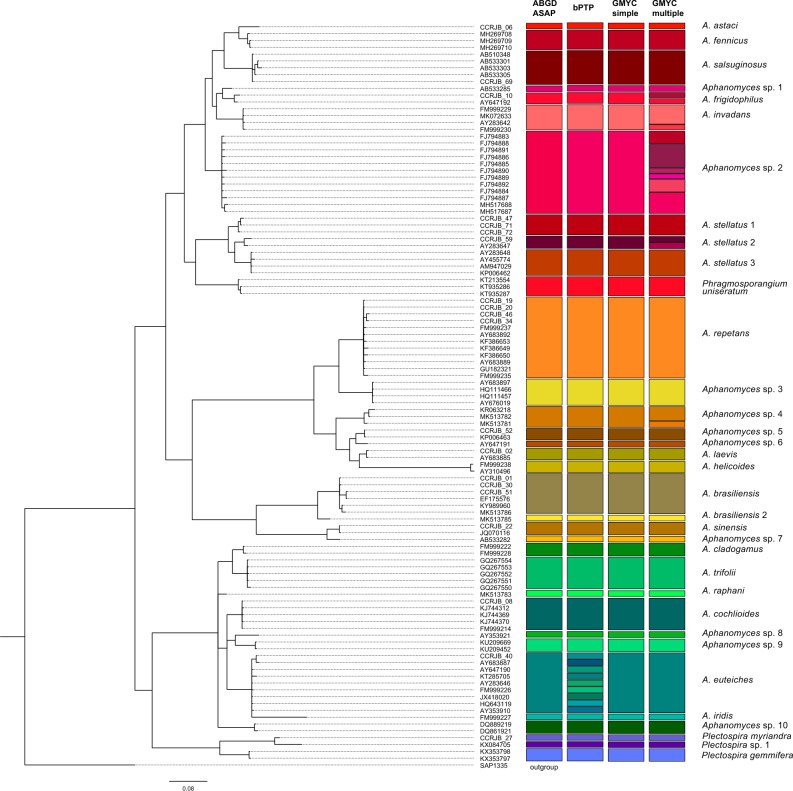
Species delimitation results of the genera *Aphanomyces*, *Phragmosporangium* and *Plectospira* based on five approaches to automatically delimit Molecular Operational Taxonomic Units (MOTUs), *i.e.*, ABGD and ASAP, bPTP, GMYC_simple_ and GMYC_multiple_, using the nuclear ribosomal Internal Transcriber Spacer (nrITS) region from 109 unique haplotypes derived from the 261-sequence matrix.

The resulting molecular database included 261 sequences of 662 base pairs. For the molecular delimitation, we included 210 nrITS sequences from the GenBank database: 201 belonged to 20 previously identified species and further unidentified sequences of *Aphanomyces*, six sequences belonged to two described species of the genus *Plectospira* and three belonged to a single species from the genus *Phragmosporangium* (Table 1). Also, we successfully amplified and sequenced the nrITS region from 51 isolates from the RJB-CSIC culture collection (Table 1). Thus, the final sequence matrix included 261 sequences, which was collapsed into 109 unique haplotypes. ABGD, ASAP and GMYC_simple_ algorithms differentiated the same number of putative species (34), and grouped the sequences congruently (Fig. 1). However, bPTP and GMYC_multiple_ approaches showed a higher number of delimited MOTUs. The bPTP approach differentiated 42 candidate species, from which 33 corresponded to the same groups differentiated by ABGD, ASAP and GMYC_simple_ (Fig. 1). However, bPTP split the species *A. euteiches* in 8 MOTUs, each one corresponding to a single haplotype. GMYC_multiple_ showed, in general, a delimitation similar to that recovered by ABGD, ASAP and GMYC_simple_. It retrieved 43 putative species, with 28 being similar to the previously mentioned approaches, whereas five candidate species differed. *Aphanomyces frigidophilus, A. invadans, A. stellatus* 2 and *Aphanomyces* sp. 4 were each separated into two candidate species. In addition, *Aphanomyces* sp. 2 was separated into six candidate species.

Given the results of the ABGD, ASAP, bPTP, GMYC_simple_ algorithms for automatic delimitation, we finally propose 34 candidate species (Fig. 1). Twenty candidate species matched with currently described taxa and 14 would corresponded to putative new species. According to all the analyses, the sequences of specimens identified as *A. stellatus* would correspond to three different species, *i.e.*, *A. stellatus* 1, *A. stellatus* 2 and *A. stellatus* 3. Similarly, sequences from *A. iridis* were identified to correspond to an independent species. Four of the potential new species were represented in the dataset by a single sequence, *i.e*., *A. brasiliensis* 2 (accession number MK513785), *Plectospira* sp. 1 (KX084705), and two *Aphanomyces* species, *Aphanomyces* sp. 6 (AY647191) and *Aphanomyces* sp. 8 (AY353921). In addition, eight other *Aphanomyces* species were detected as potential new species (Fig. 1, Table 1).

### Phylogenetic analysis

**Fig. 2:**
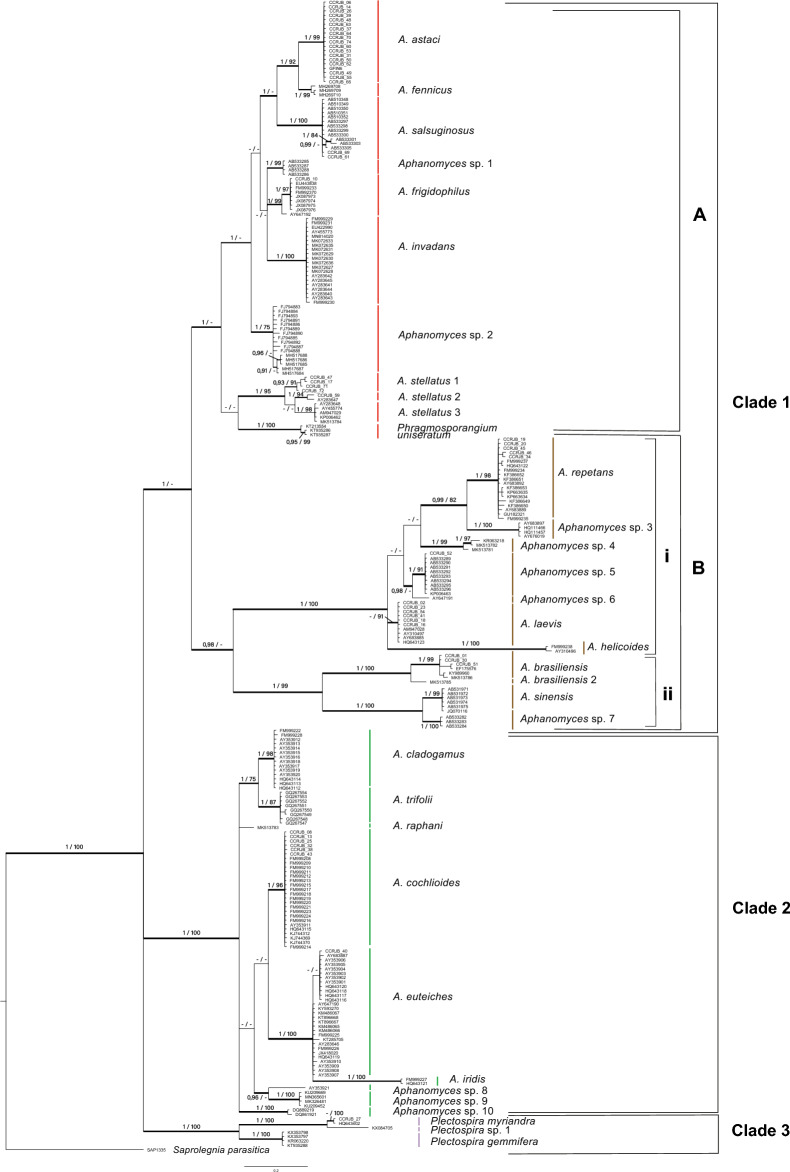
Phylogenetic tree based on nuclear ribosomal Internal Transcriber Spacer (nrITS) region of 261 sequences from the *Aphanomyces*, *Phragmosporangium* and *Plectospira* genera, obtained from 51 pure culture isolates from the Real Jardín Botánico (RJB-CSIC) and 210 sequences downloaded from the GenBank database. Values above the branches represent Bayesian posterior probabilities (< 0.95) and Maximum Likelihood bootstrap values (< 75), respectively. Scale bar indicates substitution per site. Colours within the clades make reference to the lineages identified by^10^: Clade 1A (red), corresponding to the animal parasitic lineage; Clade 1B (brown) corresponding to the saprotrophic or opportunistic lineage; Clade 2 (light green), corresponding to the plant parasitic lineage; and Clade 3 (dark green) corresponding to the plant parasitic *Plectospira* genus.

The two phylogenetic approaches (BI and ML) inferred identical topologies (represented as BI in the Fig. 2). All studied species of the genera *Aphanomyces*¸ *Phragmosporangium* and *Plectospira* formed a well-supported group (pp = 1/ bs = 100). Within this large group, three well-supported clades (1–3) are distinguished (Fig. 2).

Clade 1, supported by Bayesian inference (1), is divided into two well differentiated groups named A and B, which correspond to the animal parasitic and the saprotrophic/ opportunistic lineages, respectively, described in^10^. Within this clade, the clade A included eleven well-supported species, seven of which correspond to previously described species (*A. astaci*, *A. fennicus*, *A. frigidophilus*, *A. invadans*, *A. salsuginosus*, *A. stellatus* and *P. uniseratum*; Fig. 2). At least four of the species included in clade A have been reported as parasites (*A. astaci*, *A. invadans* and *A. salsuginosus*) or act as opportunistic parasites (*A. frigidophilus*) on freshwater animals. Two undescribed species from *Aphanomyces* were included into clade A: *Aphanomyces* sp. 1 (1/99), comprised by isolates from the icefish *Salangichthys microdon* (GenBank database), and *Aphanomyces* sp. 2 (1/75), which comprises isolates from *Daphnia* species in European lakes^46^ and in Lake Tahoe in the USA^47^. This clade also included the three-candidate species previously recognized as *A. stellatus* (Fig. 1) as well as *P. uniseratum* (1/100). All these species have been described as saprotrophic or opportunistic species and isolated from soil and water, and soil samples respectively.

Clade B was supported in the Bayesian analysis (0.98) and included nine well supported species, five of which represent previously described species (*A. brasiliensis, A. helicoides, A. laevis, A. repetans, A. sinensis*) (Fig. 2). All the species included in the clade B have been described as saprotrophic or opportunistic or found in water, except for *A. sinensis*, that was found to parasitize the turtle *Pelodiscus sinensis*. Two well-supported clades were distinguished in clade B, named (i) and (ii) (Fig. 2). The sub-clade (i) (1/100) corresponded to the saprotrophic or opportunistic lineage described by^10^ and included the species *A. helicoides, A. laevis*, and *A. repetans*, and four well supported undescribed species here named as *Aphanomyces* sp. 3, *Aphanomyces* sp. 4, *Aphanomyces* sp. 5 and *Aphanomyces* sp. 6 (Fig. 1 and 2). All of these species were described from water-related substrates, but the species *A. repetans, Aphanomyces* sp. 3 were also found in crayfish, and the species *Aphanomyces* sp. 4 in the icefish *S. microdon*. The subclade (ii) (1/99) included the recently described species *A. brasiliensis* (1/99) and *A. sinensis* (1/99), as well as the proposed undescribed taxa named as *Aphanomyces* sp. 7 (1/100) and *A. brasiliensis* 2 (1/100) (Fig. 1 and 2). The species *A. brasiliensis* 1 included previously unidentified *Aphanomyces* isolates from Ecuador in the RJB-CSIC collection among them, whereas *A. sinensis* and *Aphanomyces* sp. 7 comprised sequences associated to soft-shelled turtle *P. sinensis*^48^.

Clade 2 (1/100) corresponded to the plant parasitic lineage identified in^10^ and included eight well supported species, six previously described (*A. cladogamus, A. cochlioides, A. euteiches, A. iridis, A. raphanii, A. trifoli*), which have mostly been reported as soilborne phytopathogenic species, and three putative new taxa (*Aphanomyces* sp. 8, *Aphanomyces* sp. 9, and *Aphanomyces* sp. 10). *Aphanomyces* sp. 8 (0.96) included a single sequence associated to *Phaseolus vulgaris*, *Aphanomyces* sp. 9 (1/100) included sequences associated to *Glycine max* (Fabaceae) and *Zea mays* (Poaceae) previously identified as *A. cladogamus* and *A. cochlioides*, and *Aphanomyces* sp. 10 (1/100) included strains associated to *Phragmites australis*^49,50^.

Clade 3 (1/100) comprised individuals from the genus *Plectospira*. Two well-supported species (1/100) were recovered in this clade which corresponded to the two previously described species, *P. myrianda* and *P. gemmifera*. These species have been mostly associated to humid soils or freshwaters. However, the sequence KX084705, previously associated to *P. gemmifera*, is here identified as a proposed new species named *Plectospira* sp. 1 (Fig. 2). Overall, these species have been described as soilborne phytopathogenic, *i.e*., field soil, water bodies and reservoir sediment, similarly to the species in clade 2.

### Interaction network

**Fig. 3:**
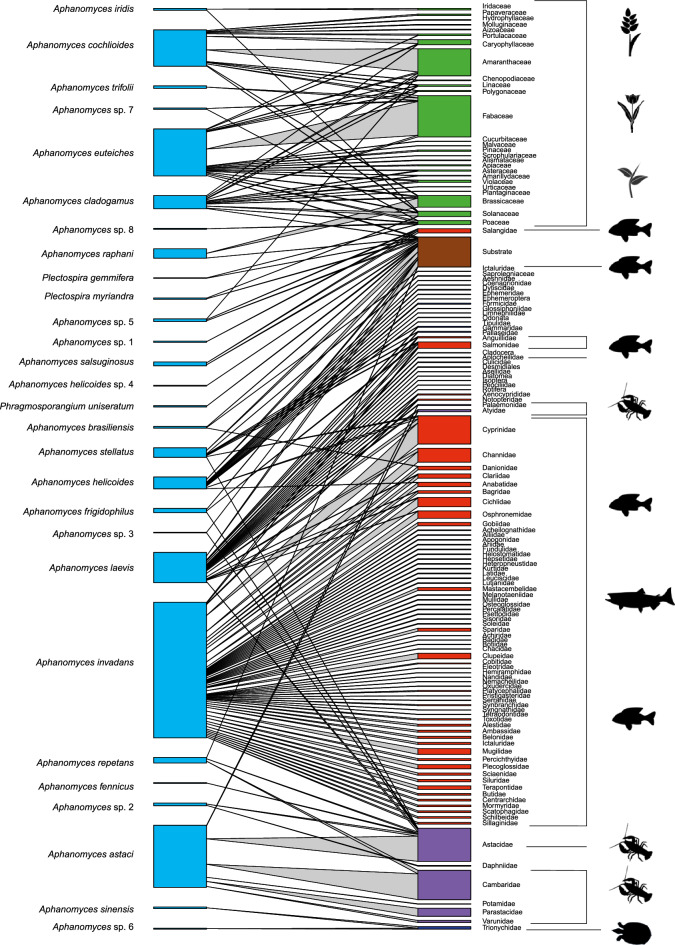
Weighted interaction network analysis in the *Aphanomyces*, *Phragmosporangium* and *Plectospira* genera (left column, blue) and their habitat preference (right column). Colours on the right column represent fishes (red), decapods (purple), plant (green) and other taxa (*i.e.*, microorganism, insects, turtle; dark blue) hosts grouped by families, and the category substrate (brown) referring to water courses, soil, cadavers and exuviae. Links are proportional to the number of citations recorded for an interaction in the literature.

The bibliographic search resulted in 1221 records from 126 scientific papers and books from 1906 to 2021 and the screening of the GenBank database (Supplementary Table). Interactions between oomycete species and their hosts are represented in the Fig. 3 (weighted matrix) and Supplementary Figure (binary matrix). In both graphs, interactions are indicated by light gray links between host families and the oomycetes species. Link thickness in Fig. 3 is proportional to the number of reports of a host belonging to that family. The left column (blue) shows 28 species (25 from the genus *Aphanomyces*, one from the genus *Phragmosporangium* and three from the genus *Plectospira*) and the right column represents the host families (plants depicted in green, fishes in red, decapods in purple, and other taxa such as microorganisms, insects or turtles in dark blue) hosts grouped by families. We also included the category “substrate” (in brown) which encompasses all oomycete reports found in water courses, soil, cadavers and unidentified insect exuviae. We included this category because of the abundant reports for some saprotrophic species that had to be otherwise excluded. In addition, we aimed to use the new proposed species names for the interaction network, but could only do it for those species whose sequences were downloaded from the GenBank database and included host information, *i.e*., for 210 records.

Network connectance was low (0.057). Both the weighted and binary matrices showed statistically significative low nestedness, although the pattern was more pronounced in the weighted matrix (wNODF: 9.482, z-score: -4.099, p-value: 0.000; NODF: 14.514, z-score: -0.3.2, p-value: 0.395). The weighted matrix showed high specialization (H_2_’ = 0.685, z-score: 10.461, p-value: 0.000). Both networks were highly modular (weighted matrix = 0.683, z-score: -13.279, p-value:0.000; binary matrix = 0.638, z-score: -41.17, p-value:0.000).

**Fig. 4:**
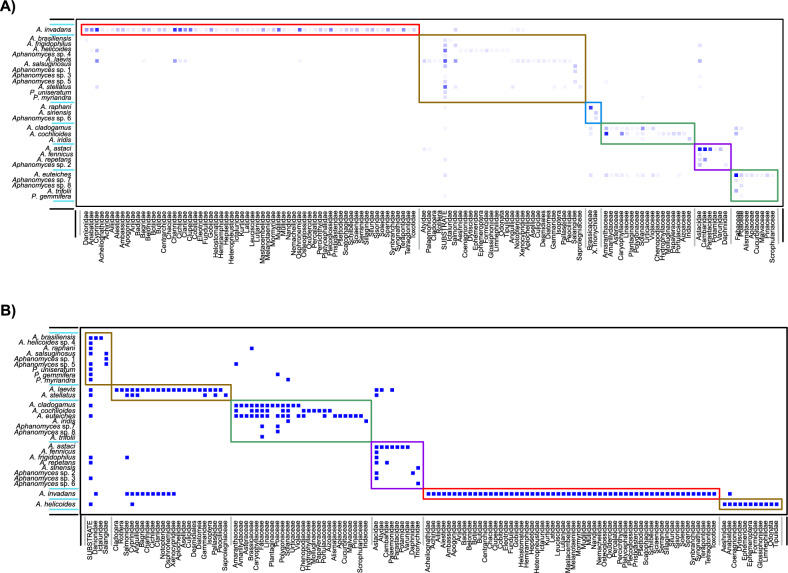
Modularity analyses for A) weighted interaction network and B) binary interaction network. The modules are identified with coloured squares corresponding to red (fish), decapods (purple), plants (green), and the category substrate (referring to water courses, soil, cadavers and exuviae) and other taxa (brown). Intensity of colours from pale to dark blue refer as the frequency of interaction between groups of species.

The modularity analysis identified six different modules for both the binary and weighted networks, but the distribution of species in these modules were different between the two approaches. In the weighted network, we identified one module including mostly saprotrophic species (Fig. 4A, in brown), two modules including three and five phytopathogenic species, respectively (Fig. 4A, in green), one module including two species associated to the Trionychidae family and one phytopathogenic species associated only to Brassicaceae (Fig. 4A, in blue), one module including species associated to freshwater Decapoda (Fig. 4A, in purple) and one module including the species, *A. invadans*, associated to freshwater fish families (Fig. 4A, in red). In the binary analysis, we identified three modules mostly associated to saprotrophic species (Fig. 4B, in brown), one module including only phytopathogenic species (Fig. 4B, in green), one module including species associated to freshwater decapods as well as some saprotrophic species (Fig. 4B, in purple), and one module including exclusively the species *A. invadans*, associated to freshwater fish families (Fig. 4B, in red). In both analyses, the only similar module was the *A. invadans* module but, overall, both analyses separated phytopathogenic species from species infecting animals into different modules, except for the case of the phytopathogenic *A. iridis* in the weighted analysis. The weighted approach could also identify two modules of species infecting animals that are not differentiated in the binary approach: (i) a module including species mostly associated to freshwater Decapoda, but also to daphnias, and (ii) the species associated to the Trionychidae family (Fig. 4). The saprotrophic species (species mostly associated to water or humid soil samples, or insect exuviae) were grouped into three different modules in the binary analysis, whereas in the weighted approach, they were mostly grouped into one module of 12 species.

We also measured the species specialization through the d’ values for the weighted matrix. Fifteen species showed low values (0.03 < d’ < 0.5), three of them showed medium values (0.5 < d’ < 0.7) and 10 showed high values (0.7 < d’ < 0.9; Table 2). All of the species exhibiting low specialization values were described as saprotrophs in soils or freshwater environments, *e.g., A. stellatus* (d’ = 0.26), A*. laevis* (d’ = 0.47), *P. gemmifera* (d’ = 0.26*), P. uniseratum* (d’ = 0.32). Many undescribed species also exhibited low specialization values, *e.g., Aphanomyces* sp. 6 (d’ = 0.03), *Aphanomyces* sp. 2 (d’ = 0.35) or *Aphanomyces* sp. 4 (d’ = 0.19). The species showing medium specialization values were the saprotrophic *A. helicoides* (d’ = 0.58), the parasitic *A. salsuginosus* (d’ = 0.56) and the undescribed *Aphanomyces* sp. 5 (d’ = 0.51). The species exhibiting the highest values of specialization are described as parasites to plants, *i.e*., *A. trifolii* (d’ = 0.70), *A. euteiches* (d’ = 0.75), *A. cochlioides* (d’ = 0.8), *A. raphani* (d’ = 0.88), *A. iridis* (d’ = 0.90), except for the undescribed *Aphanomyces* sp. 8 (d’ = 0.79); and parasites to animals*, i.e., A. invadans* (d’ = 0.89), *A. astaci* (d’ = 0.9), *A. sinensis* (d’ = 0.9), except for the undescribed *Aphanomyces* sp. 3 (d’ = 0.75). In general, the species infecting animals showed lower values of specialization compared to the species infecting plants: six out of 10 plant pathogenic species score high specialization values, compared to four out of 14 animal pathogenic species. However, the animal pathogenic species recorded some of the highest values for host specialization (Table 2).

**Table 2:** Species specialization d values in relation to other species included in the interaction network (species of the *Aphanomyces*, *Phragmosporangium* and *Plectospira* genera): d’ refers to the degree of interaction specialization in relation to other species ranging from 0 to 1; dmin refers to the minimal specialization value based on a perfect nesting of the matrix; dmax refers to the maximum theoretical value based on the observed number of interactions and marginal distributions; and d refers to the raw value of specialization for each species. We consider low values as d’ < 0.5, medium values as 0.5 < d’ < 0.7, and high values as d’ > 0.7.

	d’	dmin	dmax	d
*Aphanomyces* sp.6	0,03	1,33	6,01	5,02
*A. helicoides* sp. 4	0,19	1,67	6,41	2,57
*A. fennicus*	0,24	1,33	6,01	2,47
*A. stellatus*	0,26	0,4	3,7	1,95
*P. gemmifera*	0,26	1,67	6,41	2,9
*P. myriandra*	0,29	1,09	5,72	2,45
*A. repetans*	0,31	0,53	4,27	1,69
*P. uniseratum*	0,32	1,09	5,72	2,57
*A. frigidophilus*	0,35	0,6	4,54	1,96
*Aphanomyces* sp.2	0,36	0,68	5,02	2,89
*Aphanomyces* sp.7	0,38	1,09	5,72	2,28
*A. brasiliensis*	0,45	0,89	5,49	2,95
*Aphanomyces* sp.1	0,47	1,09	5,72	4,54
*A. laevis*	0,47	0,18	2,56	1,31
*A. cladogamus*	0,49	0,33	3,39	1,83
*Aphanomyces* sp.5	0,51	0,68	5,02	2,35
*A. salsuginosus*	0,56	0,61	4,62	2,86
*A. helicoides*	0,58	0,35	3,49	2,18
*A. trifolii*	0,7	0,68	5,02	2,25
*Aphanomyces* sp.3	0,75	1,67	6,41	1,83
*A. euteiches*	0,75	0,13	2,12	1,62
*Aphanomyces* sp.8	0,79	1,33	6,01	4,62
*A. cochlioides*	0,81	0,16	2,38	1,95
*A. raphani*	0,88	0,41	3,74	3,34
*A. invadans*	0,89	0,03	1,06	0,95
*A. astaci*	0,9	0,1	1,84	1,66
*A. sinensis*	0,9	0,89	5,49	2,02
*A. iridis*	0,9	0,74	5,31	4,85

## Discussion

This study represents the first review of host preference and specialization within the *Aphanomyces* genus, incorporating an interaction network approach to examine host–pathogen interactions within the order Saprolegniales. By reviewing the available literature and the GenBank database, we compiled a comprehensive host preference matrix containing more than 1,000 records and an updated database of nrITS sequences. This updated sequence database enabled us to delineate molecular operational taxonomic units (MOTUs) within *Aphanomyces*, *Plectospira* and *Phrasgmosporangium,* and reconstruct the life history of these groups through phylogenetic analyses. Our host–pathogen interaction analysis, framed within a bipartite network, revealed that *Aphanomyces* pathogenic species are highly specialized independently in either plant or animal hosts.

Our network analysis demonstrated that the *Aphanomyces* genus sensu lato (including species associated to *Phragmosporangium* and *Plectospira*) is a highly specialized group. The interaction network exhibited sparse connectivity, a characteristic feature of species-rich networks^51^. A typical pattern of anti-nestedness was observed, particularly in the weighted matrix, which is commonly seen in networks with high intimacy, such as those between hosts and parasites^52,53^. Similarly, the network showed a highly modular structure, another hallmark of host-parasite networks^53^. Phylogenetic tree topology distinguished two primary groups: (i) species that infect plants in wet terrestrial soils, and (ii) species that infect animals in freshwater environments. This distinction was further corroborated by the modularity of the network, which grouped most phytopathogenic species into three separate modules.

However, while phylogenetic and network analyses identified clear ecological separation between plant- and animal-pathogenic species, they did not clearly distinguish between animal pathogenic and saprotrophic species, as previously described by^10^. Instead, our analysis identified two modules of animal-pathogenic species, one specific to decapods and the other to the soft-shelled turtle *Pelodiscus sinensis*, suggesting high niche specialization for both *A. astaci* (the crayfish plague pathogen) and *A. sinensis*. In terms of species specialization, ten species showed high specialization values, including A*. astaci, A. cochlioides, A. euteiches, A. invadans, A. iridis, A. raphani, A. sinensis, A. trifolii, Aphanomyce*s sp. 3, and *Aphanomyces* sp. 8. Plant-pathogenic species generally exhibited wider host ranges and higher specialization values compared to animal-pathogenic species, which had lower to medium specialization, except for the highly specialized *A. astaci, A. invadans*, and *A. sinensis*. This pattern likely reflects the presence of numerous saprotrophic species within the group, such as *A. stellatus, A. helicoides* and *Phragmosporangium uniseratum*.

Our host preference database revealed a significant knowledge gap, especially for species infecting animals, saprotrophic and undescribed species. Many of these species lack host range data, and some have not been formally described, such as *A. repetans*^54^. To address this, future research should focus on the biology and ecology of these poorly characterized species. Studying species with broad host ranges could provide valuable insights into the mechanisms of host adaptation and specialization^12^, potentially improving our understanding of pathogen dynamics and even plague prediction^55^.

This study also highlights the critical role of molecular techniques in taxonomy when morphological characters are scarce or absent, as in *Aphanomyces*. Our MOTUs analysis confirmed 20 previously described species and identified 14 new putative species. Some of these species had been misidentified in the literature (Table 1), while others represent putative cryptic species, *e.g.*, three MOTUs assigned to *A. stellatus*. These findings underscore the need for continuous updates to molecular databases to prevent misidentifications^10,19,56^. Our definition of MOTUs lays the groundwork for further taxonomic research. Future studies should integrate culture-based data with molecular analyses to ensure accurate species identification^57^. This is particularly relevant to avoid cases of molecular characterization without official species descriptions, like the case of *A. repetans*^54^, but also to provide molecular characterization for existing species that lack molecular identification and, therefore, could not be included in the present study.

The phylogenetic results further highlight the importance of host preference in shaping the evolution of *Aphanomyces*. We confirmed the presence of lineages identified by Diéguez-Uribeondo et al.^10^, which include two major groups of saprotrophic and animal-parasitic species, as well as two lineages of plant-pathogenic species (Fig. 2). This suggests that host specialization has played a significant role in the life history of the genus^58,59^. However, our results also question the monophyly of the genus *Aphanomyces*. The positioning of sequences of the genus *Phragmosporangium* within those of the genus *Aphanomyces* (Fig. 2)^17^ suggest that the genus *Phragsmosporangium* could be evolutionary invalid, and should therefore be described as part of *Aphanomyces*, or that *Aphanomyces* could be paraphyletic. Moreover, the fact that our analysis could not resolve the phylogenetic position of *Plectospira*, which was already questioned by previous studies^16,60^ could further support the possible paraphyly of the genus *Aphanomyces*. Future studies should aim to clarify the relationships between these closely related genera and assess whether *Aphanomyces* represents a single genus or could potentially be split into two distinct genera.

In summary, our study demonstrates that the genus *Aphanomyces *sensu lato is highly specialized, with distinct lineages adapted to either plant or animal niches. By combining phylogenetic and network interaction analyses, we have established a solid taxonomic and ecological framework that will serve as a foundation for future studies on the evolutionary and ecological drivers of host specialization in oomycetes. Further research should focus on reconstructing the evolutionary histories of these species, examining virulence factors, host immune responses, and environmental variables (e.g.,^61–65^) to deepen our understanding of host specialization in *Aphanomyces*.

## Supplementary Information

Supplementary Information 1.

Supplementary Information 2.

Supplementary Information 3.

## Data Availability

Molecular data that support the findings of this study have been deposited in the GenBank repository with the following accession codes [accession codes: PX602887-PX602937 (http://www.ncbi.nlm.nih.gov/)”]. The authors declare that all other data supporting the findings of this study are available within the paper and its supplementary information files.
