# Supplementary figures and images for: Host preference and specialization in the genus Aphanomyces (Oomycetes) from molecular and interaction network insights
Source: Sci Rep. 2026 Mar 19;16:14262. doi: 10.1038/s41598-026-44513-5 (PMC13139459; doi:10.1038/s41598-026-44513-5)

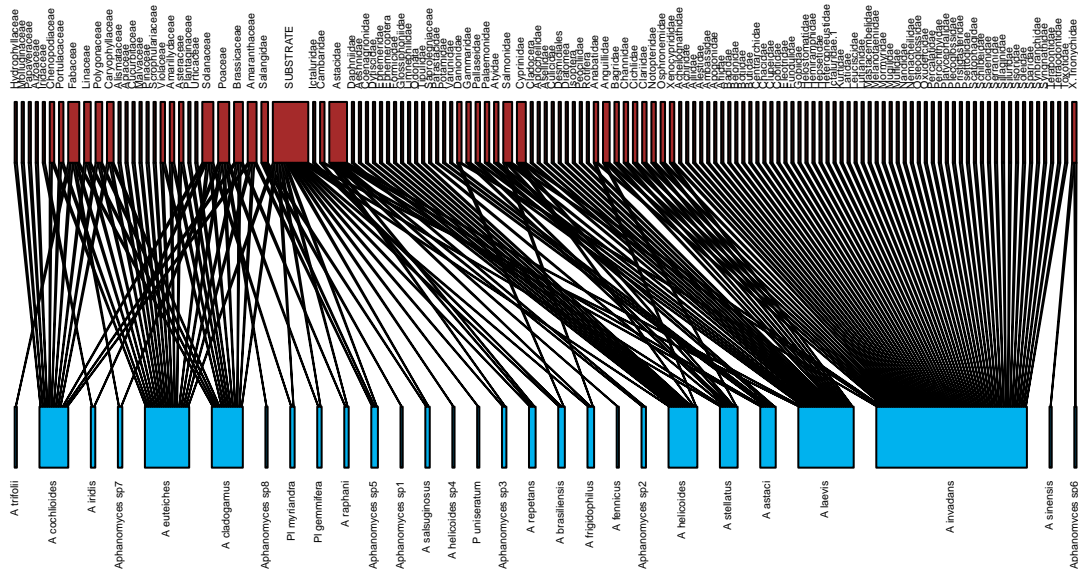

Supplement: Supplementary file 1 — Supplementary Information 1. [file 41598_2026_44513_MOESM1_ESM.pdf]
