# Supplementary material for: Host preference and specialization in the genus Aphanomyces (Oomycetes) from molecular and interaction network insights
Source: Sci Rep. 2026 Mar 19;16:14262. doi: 10.1038/s41598-026-44513-5 (PMC13139459; doi:10.1038/s41598-026-44513-5)
Supplement: Supplementary file 2 — Supplementary Information 2. [file 41598_2026_44513_MOESM2_ESM.docx]

**Supplementary information**

**Supplementary table legend**

Host-pathogen interaction database between *Aphanomyces*, P*hragmosporangium* and *Plectospira* species and their hosts recording the following information: (i) oomycete species, (ii) host or substrate of the oomycete (to the species level, if available), (iii) host family, (iv) geographical origin, (v) GenBank sequence accession if available, and (vi) bibliographic reference.

**Supplementary figure legend**

Binary interaction network analysis in the *Aphanomyces*, *Phragmosporangium* and *Plectospira* genera.
